# Water Retention Ability of the Dermis Following Polynucleotide Intradermal Injection Using a Water Distribution Model: Pilot Study

**DOI:** 10.1111/jocd.70537

**Published:** 2025-11-27

**Authors:** Jin Ho Kim, Won Seok Choi

**Affiliations:** ^1^ V&Co Cooperation Seoul Korea; ^2^ V‐Plastic Surgery Daegu Korea

**Keywords:** biophysical properties, polynucleotide, rejuvenation, transepidermal water loss

To the Editor,

Polynucleotides (PN) derived from salmon germ cells are used for skin rejuvenation. PN can scavenge free radicals, exert antioxidant activity, and influence fibroblast metabolism, thereby promoting the renewal of various dermal components [1]. PN readily binds to water molecules in the dermis. Increased water retention in the dermis enhances skin viscoelasticity, improves structural integrity, and increases firmness [2]. Quantifying the extent of water binding within the dermis when using PN remains challenging. In this letter, we propose a water distribution model that spans the dermis to the stratum corneum and characterizes dermal water retention that is based on measurable biophysical properties of the skin.

There are three layers of hydration: stratum corneum (SCH), intra‐epidermal (EH), and intra‐dermal (DH) (Figure 1). According to Uehara et al. transepidermal water loss (TEWL) is proportional to the water gradient between EH and SCH and is inversely proportional to the thickness of the stratum corneum [3]. We assume that EH is maintained through a specific distribution received from DH and can be represented as EH = *β* × DH, where *β* is the water retention ability coefficient (Equation 1). Detailed information for the equation is offered in Data S1.

(1) $$\beta =\frac{Y}{M}\times \left(\mathrm{SCH}+\text{TEWL}\frac{T^{\gamma }}{k}\right)$$

where *Y* = Young's modulus, *T* = epidermal thickness, *M* and *k*, and γ is a constant.

**FIGURE 1:**
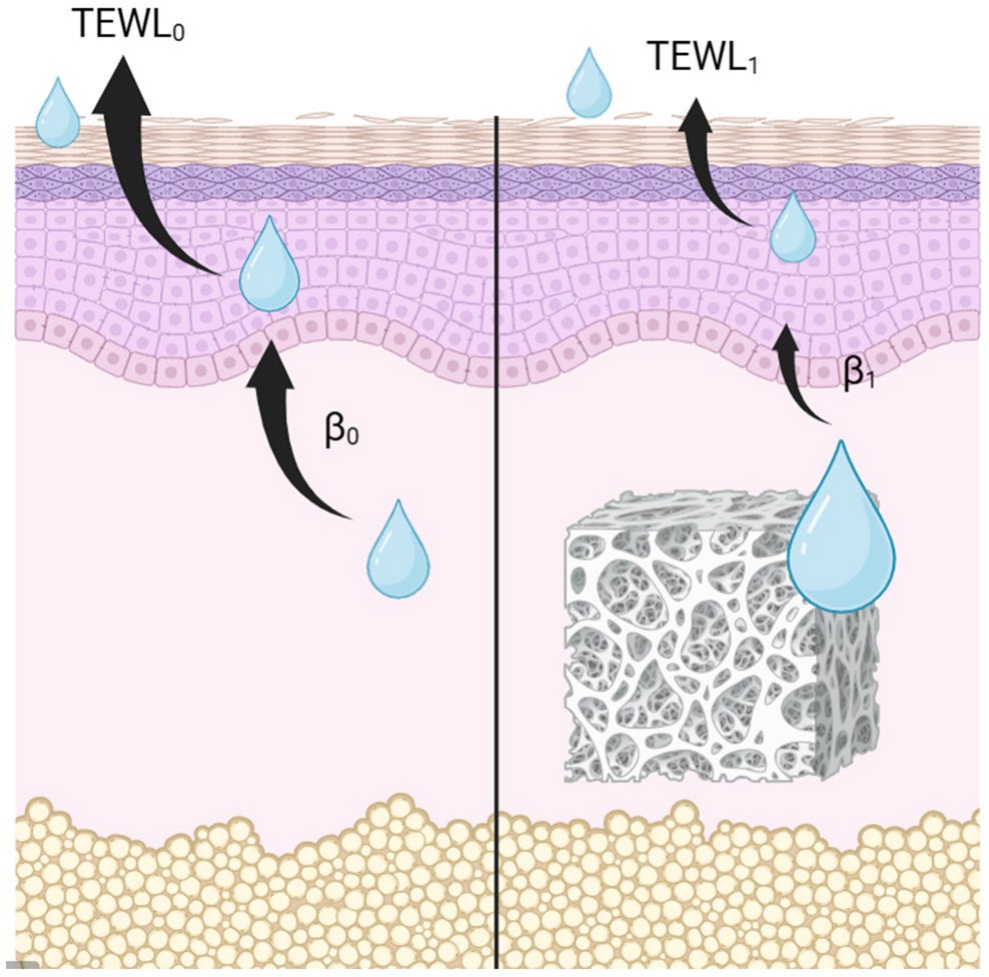
Distribution of water in three layers. (A) before and (B) after injecting polynucleotide (PN) in the dermis. Water retention ability coefficient (*β*_0_ and *β*_1_) could be changed after polynucleotide was injected in the dermis. Fewer water would be distributed to the epidermis and induce lower transepidermal water loss (TEWL), since the gradient of water between the epidermis and stratum corneum became lower.

A study approved by the Korean Public Institutional Review Board was conducted on eight participants with healthy skin, in which injections were administered at 1‐cm intervals on both cheeks. Normal saline was injected on the left side, Rejuran (Pharma Research Products Inc., Seoul, Korea), a device filled with a transparent liquid consisting of 20 mg/mL PN, was injected on the other side. Before and 2 weeks post‐injection, several skin biophysical properties were measured, including conductance, TEWL, gross elasticity (R2), Young's modulus (Y), and dermal thickness [4, 5]. Detailed methods and limitations of the study are described in Data S2.

A substantial decrease in *β*_1_ compared to *β*_0_ was observed, where *β*_0_ and *β*_1_ are the *β*‐values prior to, and 2 weeks after, PN injection (Figure 2A,B). PN injections promoted greater water retention within the dermis, thereby reducing the amount of water distributed to the epidermis. PN enhanced viscoelasticity by increasing water retention, leading to a reduction in Y, which indirectly lowered TEWL by reducing the water gradient from the epidermis to the stratum corneum. When the initial *β* value was high—reflecting poorer skin condition—the PN treatment induced a more pronounced change in *β*, demonstrating this relationship with high accuracy. Therefore, measuring *β*, a function derived from skin biophysical parameters, can predict the potential efficacy of PN treatment for individual patients. This *β* function is expected to be useful for comparing the effects of various skin boosters and predicting the short‐term efficacy of skin boosters.

**FIGURE 2:**
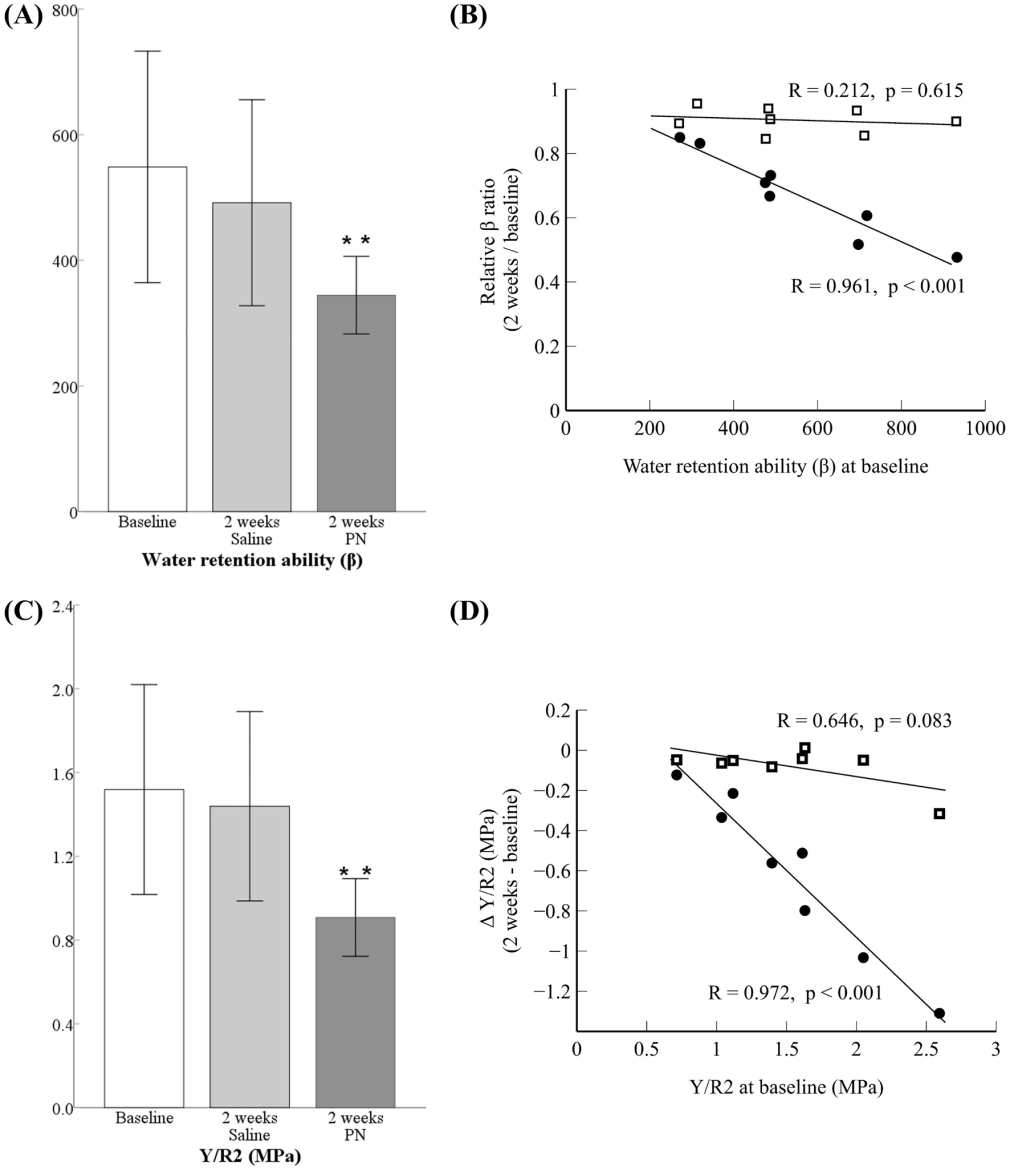
(A) Change of water retention ability coefficient (*β*) after saline injection and polynucleotide (PN) dermal injection. (B) relative ratio of *β* (after/before). PN injection showed change of dermal water retention ability after PN injection (black circle), while saline did not showed change of dermal water retention ability (white box). A higher initial β leads to more changes in β after polynucleotide dermal injection. (C, D) Pre‐treatment value of Young's modulus over R2 (gross elasticity) and differences between pre‐ and post‐treatment values. PN injection showed change of dermal elasticity after PN injection (black circle), while saline showed little change (white box). **p < 0.01.

Moreover, poorer initial skin condition was associated with greater differences between pre‐ and post‐treatment values, making the Y/R2 ratio a useful function for evaluating efficacy. Because an increase in Y corresponds to reduced skin elasticity and a decrease in R2 indicates diminished elasticity, a larger Y/R2 ratio implies lower skin elasticity. The difference between pre‐ and post‐injection Y/R2 illustrates that individuals with poorer baseline elasticity experienced greater improvement following treatment (Figure 2C,D). The development of such standardized functions could serve as a foundation for artificial intelligence‐based predictive models, enabling the evaluation of treatment efficacy and suitability [6].

In summary, we used a water distribution model to analyze the changes in skin biophysical properties after PN injection. The observed variations in *β* and Y/R2 clearly indicate that poorer initial skin conditions correspond to more substantial *β* and Y/R2 changes, suggesting that *β* and Y/R2 could serve as a predictive function for treatment outcomes.

## Author Contributions

**Jin Ho Kim:** contributed to data curation and analysis, investigation, and writing the original draft. **Won Seok Choi:** contributed to the conceptualization, validation, and reviewing of the original draft.

## Ethics Statement

This study was approved by the public Institutional Review Board designated by the Ministry of Health and Welfare of Korea. All participants provided written informed consent.

## Conflicts of Interest

The authors declare no conflicts of interest.

## Supporting information


**Data S1:** jocd70537‐sup‐0001‐Supinfo.docx.

## Data Availability

The data that support the findings of this study are available from the corresponding author upon reasonable request.
