# Supplementary material for: Water Retention Ability of the Dermis Following Polynucleotide Intradermal Injection Using a Water Distribution Model: Pilot Study
Source: J Cosmet Dermatol. 2025 Nov 27;24(12):e70537. doi: 10.1111/jocd.70537 (PMC12658717; doi:10.1111/jocd.70537)
Supplement: Supplementary file 1 — Data S1: jocd70537‐sup‐0001‐Supinfo.docx. [file JOCD-24-e70537-s001.docx]

**Supplementary 1.**

There are three layers of hydration: stratum corneum (SCH), intra-epidermal (EH), and intra-dermal (DH) (Figure 1). According to the theory of transepidermal water loss (TEWL) proposed by Uehara et al., TEWL is proportional to the water gradient between EH and SCH and is inversely proportional to the thickness of the stratum corneum (Eq. 1):^3^

$TEWL=(EH-SCH)\times\frac{k}{T^{\gamma}}$ (1)

where k is a constant, γ is a coefficient, and T is the thickness of the stratum corneum.

We assume that EH is maintained through a specific distribution received from DH and can be represented as EH = β × DH, where β is the water retention ability coefficient:

$TEWL=(\beta DH-SCH)\times\frac{k}{T^{\gamma}}$ (2)

According to Kalra et al., of the various factors that influence the Young’s modulus of the skin, Langer’s lines, age, and skin thickness can be assumed to yield nearly identical values when measured at the same anatomical site.^4^ Although DH cannot be directly quantified, it has been hypothesized that an increase in DH leads to a decrease in Y:

$TEWL=(\beta\frac{M}{Y} -SCH)\times\frac{k}{T^{\gamma}}$ (3)

Where M is constant, rearranging for β gives Eq. (4):

$\beta=\frac{Y}{M}\times\left( SCH+TEWL\frac{T^{\gamma}}{k} \right)$ (4)

**Supplementary 2.**

A study approved by the Korean Public Institutional Review Board was conducted on eight participants with healthy skin, in which injections were administered at 1-cm intervals on the left side. Rejuran (Pharmaresearch Products, Inc., Seoul, Korea), a device filled with a transparent liquid consisting of 20 mg/ml PN, was used in this study. Before and two weeks post- PN injection, several skin biophysical properties were measured, including conductance, transepidermal water loss (TEWL), gross elasticity (R2), Young’s modulus (Y), and dermal thickness. Biophysical measurements were performed under standardized room temperature (20–21 ℃) and humidity (40–50% relative humidity). A conductance pin probe, TEWL X Probe, suction-cup chamber, and high resolution ultrasound imaging (Dermalab® Combo 4, Cortex Technology, Hadsund, Denmark) were used for measuring conductance, TEWL, R2, and dermal thickness, respectively. All measurements were taken at the point where the vertical line from the lateral canthus intersected the horizontal line connecting the nasal ala and tragus. As a pilot study, this research has some limitations, including a small sample size and restricted age and sex diversity.

The elastic plate model could be used to calculate Young’s modulus:^5^

$Y=0.3125\frac{\Delta p}{\Delta\mathrm{wh}^{3}} (MPa)$ (5)

where E = Young’s modulus, w = deflection of the skin surface, p = suction pressure, and h = skin thickness measured using high resolution Dermalab® ultrasound imaging.

**Supplementary 3.**


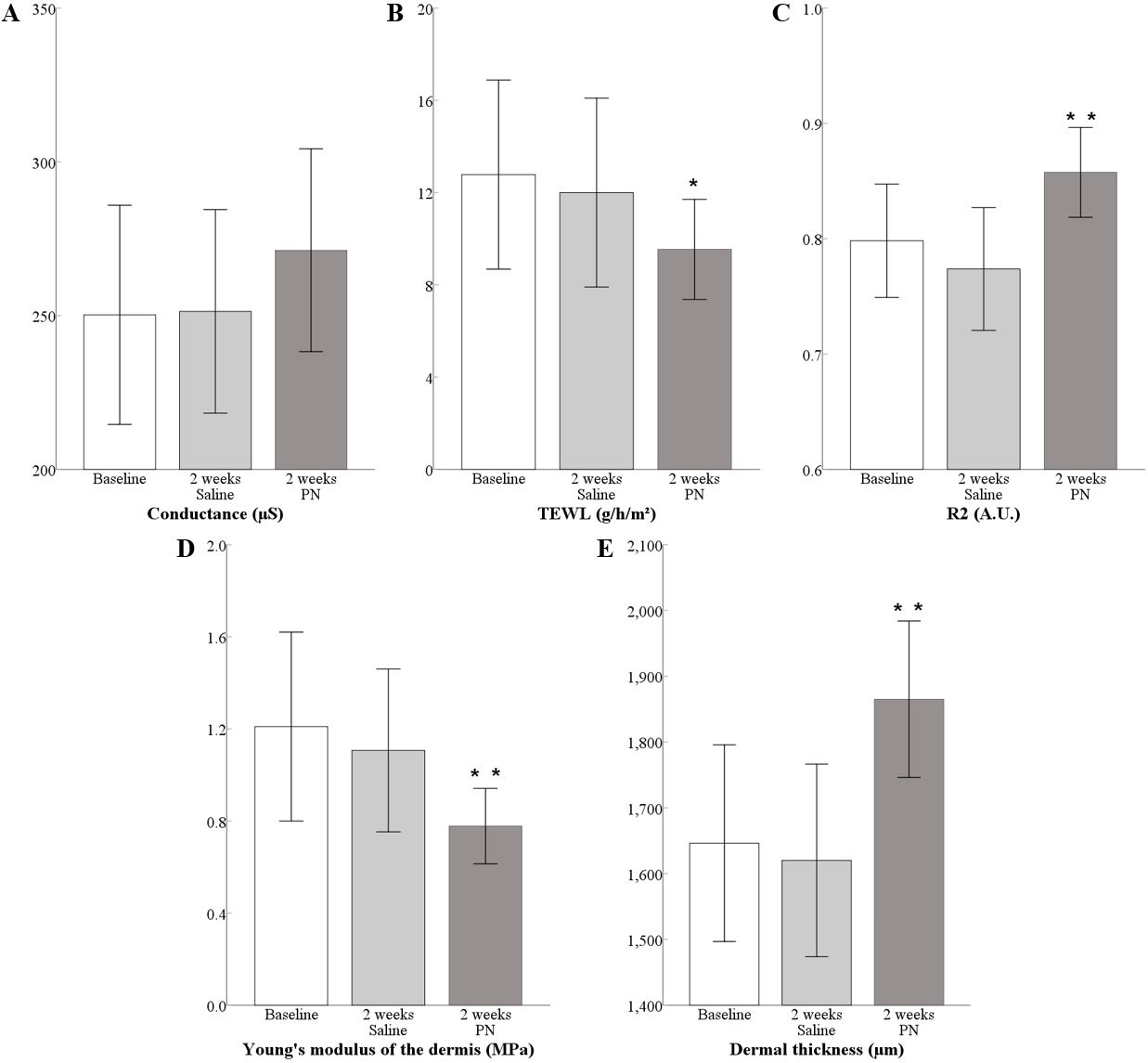


**Figure 2. Comparison of measurable skin biophysical properties of the skin before, 2 weeks after saline injection, and 2 weeks after polynucleotide injection; (A) Conductance (B) Transepidermal water loss (TEWL) (C) R2 (gross elasticity) (D) Young’s modulus of dermis (E) Dermal thickness. *p<0.05, **p<0.01**

The SCH value did not show substantial changes, whereas TEWL decreased (Figure 2). Skin elasticity (R2, Y, and dermal thickness) improved substantially.

**References**

1. Park KY, Seok J, Rho NK, et al. Long-chain polynucleotide filler for skin rejuvenation: efficacy and complications in five patients. Dermatol Ther. 2016;29:37-40.

2. Lee YJ., Kim HT., Lee YJ., et al. Comparison of the effects of polynucleotide and hyaluronic acid fillers on periocular rejuvenation: a randomized, double-blind, split-face trial. J Dermatolog Treat. 2022;33(1):254-260. doi:10.1080/09546634.2020.1748857

3. Uehara O., Kusuhara T., Nakamura T., Transepidermal Water Loss Estimation Model for Evaluating Skin Barrier Function. Adv Biomed Eng. 2023;12:1-8 https://doi.org/10.14326/abe.12.1

4. Kalra A, Lowe A, Jumaily AA. An overview of factors affecting the skin’s Young’s modulus. J Aging Sci. 2016;4(2):1000156. doi:10.4172/2329-8847.1000156

5. Sutradhar A, Miller MJ. In vivo measurement of breast skin elasticity and breast skin thickness. Skin Res Technol. 2013;19(1):e191-e199. doi:10.1111/j.1600-0846.2012.00627.x

6. Thunga S, Khan M, Cho SI, et al. AI in aesthetic/cosmetic dermatology: current and future. J Cosmet Dermatol. 2025;24(1):e16640. doi:10.1111/jocd.16640
